# An Edge-Supported Blockchain-Based Secure Authentication Method and a Cryptocurrency-Based Billing System for P2P Charging of Electric Vehicles

**DOI:** 10.3390/e24111644

**Published:** 2022-11-12

**Authors:** A. F. M. Suaib Akhter, Tawsif Zaman Arnob, Ekra Binta Noor, Selman Hizal, Al-Sakib Khan Pathan

**Affiliations:** 1 Department of Computer Engineering, Sakarya University of Applied Sciences, Serdivan 54050, Sakarya, Turkey; 2 Department of Mechanical and Production Engineering, Islamic University of Technology, Gazipur 1704, Bangladesh; 3 Department of Computer Science and Engineering, United International University (UIU), Dhaka 1212, Bangladesh

**Keywords:** blockchain, cryptocurrency, edge computing, electric vehicles, Ethereum, P2P charging

## Abstract

The popularity of electric vehicles (EVs) is constantly increasing, as they use relatively greener, sustainable energy. However, it is a fact that the charging stations for EVs are yet to meet the demand. It could be a great solution if a peer-to-peer (P2P) charging system could be initiated by anyone who wants to make their garage’s charge points publicly available for commercial purposes, named a home charging station (HCS). In this work, our idea is to bring interested charging stations under a network of nodes and a blockchain-based management system, where the blockchain is responsible for ensuring the authenticity of both the charging stations and charge receiver. A cryptocurrency-based payment system has also been proposed to ensure transactions’ security, integrity, transparency, and immutability. A reputation management system is applied to maintain the quality of service. Miners with high processing power are used to alleviate lagging during block creation, supported by edge servers. The proposed system has been implemented by using virtual machines. A theoretical analysis is presented to assess the compatibility and possible cost requirements to implement the system in a real-world scenario.

## 1. Introduction

Vehicles with the potential of using renewable energy sources, such as electric vehicles (EVs), have caught worldwide attention in recent times [1,2,3]. These vehicles do not depend on fossil fuels but use other renewable energy sources to reduce gas emissions. As stated in [4,5], by the year 2040, it is projected that renewable energy is to come to equivalence with coal and natural gas-based electricity generation. Additionally, the EV stock is expected to reach at least 140 million by 2030. However, it will take a long period of time to integrate them efficiently within the infrastructure. At present, EV users are hesitant to use their vehicles for long drives, and potential customers go through the dilemma of choosing an EV over a traditional vehicle, as there are so few charging stations. Level 2 charging equipment can provide a vehicle with 10 to 20 miles of range for every hour of charging. With the necessary set-up, anyone can make their garage available to charge an EV for long-distance traveling [6]. This could provide an abundance of charging stations across the country within the existing infrastructure. Our idea in this setting is that the EVs and the home charging stations would be two different party nodes under a secure and reliable system with availability, security, preservation of privacy, and payment facilities.

Malicious operators can seriously threaten EVs’ security and privacy through various malicious exploitations [7,8,9], e.g., privacy leakage, falsification, node impersonation, or advertising fraudulent charging services. To provide secure charging services for EVs, many innovative mechanisms have been proposed so far, and some are even implemented to some extent [10,11]—e.g., trust mechanism and monetary approaches. However, the trust mechanism is not sustainable and susceptible to Sybil attacks and whitewashing attacks, and the monetary approach relies on trusted centers. Trusted centers may not only leak users’ private information for profit, but also may be vulnerable to attacks. In this context, blockchain [12] offers a unique platform for secure energy transactions within a distributed network without trusted agents through the use of an immutable ledger, cryptocurrency, and the execution of smart contracts.

As we know from various recent works and interest shown by a wide range of researchers, blockchain technology can come in handy for various management systems. A blockchain is a decentralized, distributed, open ledger, and each node in the network has a copy of the ledger. It was developed as a peer-to-peer network without third-party intervention [13]. The blockchain’s integrity is based on strong cryptography and hash functions that provide validation and chain blocks together on transactions, making it nearly impossible to tamper with a block or any individual transaction without being detected [14].

As the number of online systems has increased, we have witnessed that the threats of various types of cyber attacks have also increased significantly. Among the various types of attacks, unauthorized entities or malware-based attacks can cause fatal damage to the system [15]. Thus, a deficiency in the proper authentication process can make a P2P system vulnerable to various types of attacks. In our proposed system, a blockchain-based authentication system is used, so that before making an agreement, the entities (EVs and the HCS) can check the authenticity of each other. Again, while getting services from the HCS, a proper charging measurement system is essential to calculate the amount of the electricity that has been exchanged from the HCS to the EV. Moreover, it is also required to determine the number of bills to be paid. In our system, a smart meter is used to calculate the amount of charging, and the HCS would share that by using the blockchain.

Due to the popularity of cryptocurrency in the financial sector, researchers have also started utilizing it in various fields. In fact, the transparency, trustworthiness, worldwide availability, convenient exchange facilities, ease of access, minimum transaction cost, etc., encourage the business world to utilize cryptocurrency [16,17]. Hence, in this study, a cryptocurrency-based payment system has been used for the system: after calculating the amount (to be paid), the smart meter requests a transaction in the blockchain. The system will automatically deduct the amount from the EV, which will be credited to the HCS’s account. After each transaction, the service receiver, i.e., the EV owner, can provide feedback about the service received. It will help the server suggest that HCS out of those nearby, as an HCS with a higher rating will come before one with a lower rating. As generating blocks for a blockchain requires high computational power, edge computing services have been used to perform the complex mathematical calculations required for mining.

In this work, we used a combination of multiple protocols, and the main contributions of this can be summarized as follows:

- A blockchain-based electric vehicle charging management system is proposed where an EV can receive charging (or recharge) when necessary from anywhere in the world. HCSs from anywhere in the world can join the network and can earn money by providing charging services to EVs. Management, searching, etc.—related services—would be provided by the blockchain.
- To avoid unauthorized access, malware, DDoS (distributed denial-of-service), or any other security attacks, a blockchain-supported authentication system is employed. Additionally, the system also preserves the privacy of the members.
- To remove confusion, miscalculations, etc., a smart billing system is proposed in this paper where two different agents are responsible for measuring the amount of charge exchanged between EVs and HCSs. Moreover, to avoid the hassle of payment, the smart billing system (SBS) automatically calculates the amount to be paid and creates a transaction in the blockchain after the charging process is finished.
- To ensure safe, secure, transparent, and authentic payment, a cryptocurrency-based payment system is proposed, which was developed by using the Ethereum blockchain. The payment system is handled by the blockchain, and currency transfers will happen automatically.
- To ensure the transparency and quality of service (QoS), a reputation management system is also proposed, where EVs have the opportunity to express the grade of the service received.
- The system was developed using the Ethereum blockchain, with which the authentication, billing, payment, and reputation management system were simulated.

To explain the full system, the paper is organized as follows: Section 2 presents the motivations for the proposed system, together with notable research. Section 3 gives some preliminary knowledge on the issues and definitions that could help the general readers get useful information, and it establishes the importance of this work. Section 4 describes the system’s architecture with its components and transactions. The implementation details are provided in Section 5. Then, Section 6 contains the performance analysis, and in Section 7, challenges and limitations are discussed. The paper concludes with Section 8 with future research directions.

## 2. Related Work and Motivation

The energy-sharing method is not new, yet the popularity of using EVs has unlocked a vast area of research. In this section, we will present some previously published energy-sharing-related methods.

In [18], Zhang et al. depicted a typical incentive-based approach in the smart grid environment and explored vehicle-to-vehicle (V2V) scenarios. This is a cloud-based energy trading process with a contract theory approach. Tushar et al. introduced an incentive game-based mechanism for distributed renewable energy management in a smart community [19] to improve the operator’s profit and minimize total energy trading cost. Bera et al. [20] introduced a novel cooperative energy consumption system within communities in the smart grid to mitigate energy consumption costs for users and reduce the peak-to-average ratio. In [21], a global control scheme is proposed for electric energy micro-storage systems in smart communities to improve the local power quality of demanded and current power consumption globally.

Sharing energy between two peers requires ensuring the security services, including authenticity, privacy, integrity, attack prevention capability, etc. To provide these, in [22], a token-based decentralized energy trading system was shown that enables peers to perform transactions anonymously and securely. The system was developed by using multi-signature and anonymous encryption methods. Li, Z. et al. [23] provided a secure distributed energy trading market and designed a novel energy blockchain system in the industrial Internet of things (IIoT) environment. They implemented the system by using a consortium blockchain. Li, L. et al. [24] presented a novel announcement network named credit-coin that uses blockchain technology to protect vehicles’ privacy and motivate users to broadcast traffic information. In [25], a pragmatic blockchain utilization case is introduced for machine-to-machine (M2M) transactions of energy within the housing society environment. Based on the lightning network and smart contract in the energy blockchain ecosystem, Huang et al. presented a decentralized security model for the enhancement of the security of trading between EVs and charging piles in the peer-to-peer (P2P) network [26].

A secure way to pay and proper pricing need to be ensured for a P2P EV charging system. To do that, Zou et al. designed a progressive second-price auction game mechanism for resolving large-scale EV charging cooperation problems. They have ensured incentive compatibility over a finite horizon in their work [27]. Mohammadi et al. depicts a distributed cooperative charging scheme for plug-in electric vehicles (PEVs) to minimize the charging cost for PEV fleets with the integration of a receding horizon method [28]. Liu et al. [29] proposed a novel renewable energy pricing scheme for smart communities to reduce the total electricity bill of the residential users utilizing an advanced cross-entropy optimization method in smart home energy scheduling. A contract game-based direct-energy trading system is proposed by Zhang et al. [30] for modeling the decision-making process of electricity operators and consumers in vehicular edge computing networks. Yang et al. [31] presented a coordinate EV charging mechanism in a microgrid-powered setting via wind-powered generators through a Markov decision process (MDP) approach. Utilizing stochastic dynamic programming methods, Wu et al. [32] proposed smart-home energy management integrated with PEVs (plug-in electric vehicles) to address the problem of intermittent renewable energy supplies to minimize the electricity cost.

With the popularity of blockchains, some other projects have been found where a blockchain is primarily utilized for EV charging. For example, in [33], a blockchain was proposed to ensure a secure and trusted electricity trading solution. The authors of [34] utilized blockchain to create a trusted distributed environment for charge sharing. In [26], a blockchain was used for charging management, and in [7], it was used to store the trading records between EVs and charging stations.

A two-stage autonomous EV charging coordination method implemented on blockchain was shown by Ping et al. [35] to enable dependable EV charging coordination in the absence of a third-party coordinator. This mechanism also preserves the privacy of the users. Wang et al. developed an optimization model on a blockchain framework to manage the operation of crowdsourced energy systems (CESs) with peer-to-peer (P2P) energy trading transactions (ETTs) [36]. One of the new paradigms created by the decarbonization, decentralization, and digitalization of the energy supply chain (that enables direct exchange between energy users and producers) is depicted in [37]. Chen et al. proposed an energy trading framework that marries a blockchain and distributed optimization; the blockchain enables checks and balances among the participants and disables dishonesty [38].

Consider the above-mentioned papers. Our take is that they provide partial solutions in terms of P2P charge-sharing systems. A model is required where there will be secure communication and management, easy and time-saving payment facilities, and reliable quality of service. Hence, a complete solution is proposed where all the required features are available for the users. Additionally, in almost all of these previous work, there was no implementation to show the compatibility and validity of their approaches. Hence, there is indeed a gap in the existing literature. We brought forth a real-world implementation by using Ethereum blockchain to show our system’s compatibility, to understand the behavior of the components and responses, and to collect important data from the system. The motivations for this work are presented in the next section.

### Motivation

Electric vehicles are popular nowadays, and many are embracing the idea of an electricity-run car with the utmost interest. However, in reality, EV owners are still not confident enough or are often hesitant to go for long road trips. This is mainly due to the relatively small number of available charging stations compared to conventional fuel stations. If the car has a dual mode that runs both on electricity and traditional fuel, the problem is making a decision of how much fuel is to be always carried (how frequently the fuel tank needs to be filled up) and how frequently to charge the battery of the EV. This adds an extra layer of decision making for EV owners. In the usual case, an owner would like to run the vehicle on electricity, as it is designed to run in that way, which makes it often significantly costlier than many other regular cars. Thus, the issue we have here is that we need some kind of efficient charging mechanism or model to support long journeys by EVs. Some previous works tried to solve this issue in some ways, but as mentioned in the previous section, almost all of them fall short of the required efficiency, and some have not even given enough thought to this issue; i.e., models are available, but they are not comprehensive or advanced enough. We found a gap in the existing literature on this issue, and that basically motivated us to devise our mechanism.

P2P systems can be used for this issue of charging, but they would require trust and security, as complete strangers can come to a charging station. In this case, a good way to think about getting a solution is that anyone who is registered under the blockchain would be allowed to get the service. The blockchain makes the system secure and trustworthy, preventing malicious attacks while recording each transaction. This would create an increase in the number of charging stations throughout a country, creating a source of income for charge-point providers without the intervention of a third party.

Furthermore, it can reduce the pressure on expanding the infrastructure for setting up new charging stations to meet the rising demands of EV charging. This P2P charging environment will enable an opportunity to see an increase in the frequency of EVs in sectors such as delivery and medical (ambulance), where EVs are still considered inefficient. As we aim to move on to a greener future, we need to exploit our infrastructure the most we can without consuming more areas to set up charging stations.

To minimize the hassles such as platform dependency of the payment system, uncontrolled pricing, and delays because of payment confirmation, in the proposed method, all those issues are solved by using blockchain-based cryptocurrency. A charging management system is responsible for measuring the transferred energy, and it calculates the price according to that. Then, the system can directly make a transaction in the blockchain, and the amount will be automatically deducted from the service receiver’s account. In this way, the payment will be much easier, easy to handle, and time-saving for both parties.

A reputation management system is added to the proposed method where EVs can provide a review about the service received, more specifically, about the HCS. Upon receiving a review, the server will calculate the mean of overall ratings for each HCS, and while suggesting nearby HCSs to the EVs, HCSs with relatively higher ratings will get priority on the list. Moreover, as the HCSs have the authority to fix their own pricing, they can balance the ratings and pricing.

## 3. Prior Knowledge

### 3.1. Blockchain

**Figure 1:**
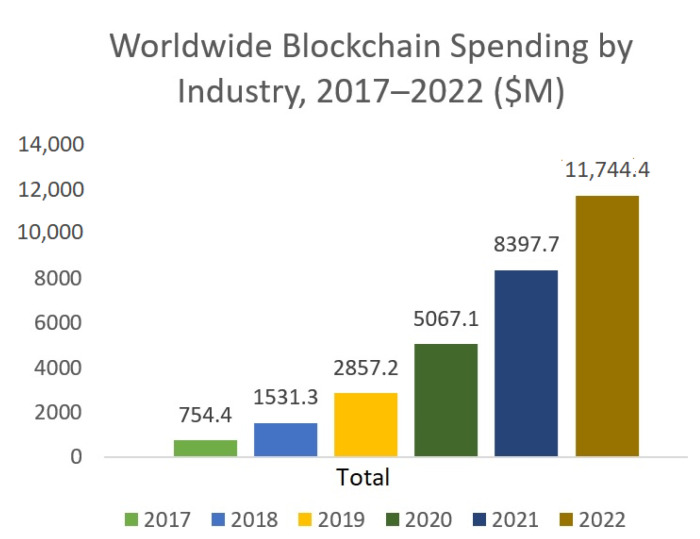
Worldwide blockchain spending by industry.

It can be strongly said that blockchain is the future of secure currency management. Security, integrity, worldwide availability, preservation of privacy, immutability, transparency, etc., are the basic services provided by blockchains [39]. Distributed storage and decentralized storing system are considered as additional advantages. To understand the popularity, the amounts of money invested (over the years) by different industries for blockchain are presented in Figure 1 (adopted from [40]).

Protection from several types of attacks is another special feature of blockchains. Due to its decentralized and distributed storage technique, many typical attacks, including Sybil attacks, unknown source attacks, man in the middle (MITM) attacks, unauthorized entry, and DDoS (distributed denial-of-service), are not possible to perform on a blockchain. Additionally, consensus protocols put on another level of security on this, which can ensure the integrity and stability of the information.

### 3.2. Ethereum

Ethereum was selected as the blockchain for the proposed method, which brings several advantages to the system. Although in terms of popularity, Ethereum loses first position to Bitcoin, it still has some very exclusive features that make it a popular choice for industries. The most relevant feature of Ethereum is the smart contract [41], which makes it a digital asset management system rather than just a money transfer system. Due to smart contracts, it is possible to manage the full system by using one platform, which is not possible for other types of blockchain, such as Bitcoin, Zcash, Dash, Peercoin, Ripple, Monero, and Multichain.

Ethereum supports more transactions per second than most of the other blockchains; again, Ethereum does not have any coin limit. On the other hand, while most of the blockchains support the latest scripting language by Bitcoin called Bitcoin Script [42], Ethereum supports multiple languages that are similar to the most popular languages. Examples include Solidity, which is similar to Java Script and C; Serpent, which is similar to Python; and LLL, which is similar to Lisp. Table 1 shows the advantages of using Ethereum over other blockchains.

### 3.3. Cryptocurrency

Cryptocurrency is a digital currency secured by cryptographic algorithms, and it provides high security, availability, transparency, etc. It can be said that cryptocurrency is the future of the economic world. In the proposed method, the payment system is managed by using *Ether*, which is a cryptocurrency supported by Ethereum blockchain. It is available all over the world, decentralized, cost-effective, time-saving transaction-wise facility, and self-governed; and its convenient money-exchange facilities make it one of the biggest currencies in the world. The use of *Ether* in the proposed method makes the payments automated, secured, easily accessible, time-saving, and hassle-free. In particcular, the EV does not have to wait after receiving charges, as the SBS automatically sends the billing information and makes the payment.

### 3.4. EDGE Computing

Due to consensus management, calculating complex cryptography and hash functions requires time to generate a block in the blockchain. On the other hand, as the proposed method would require mobile services, the computational workloads would be handed over to the edge computing servers. In fact, today’s high-speed Internet, such as 5G and the upcoming 6G, will be highly efficient for accessing large amounts of data from edge servers. Thus, rather than arranging and spending a huge amount of money on a physical server setup, utilizing an edge server is proposed in this system.

## 4. System Structure

In this paper, a complete solution for an EV charging system is proposed. The system is comprised of three main protocols, which are: (1) The authentication protocol, (2) the smart billing system, and (3) the reputation management system. In this section, firstly, there are short descriptions of the components of the system, followed by detailed information about the communication, authentication, billing, and reputation management system.

### 4.1. Components

Four main components participate in this system. These are:

– Electric vehicles (EV).
– Home charging stations (HCS).
– Smart billing system (SBS).
– The blockchain and edge servers.

**Figure 2:**
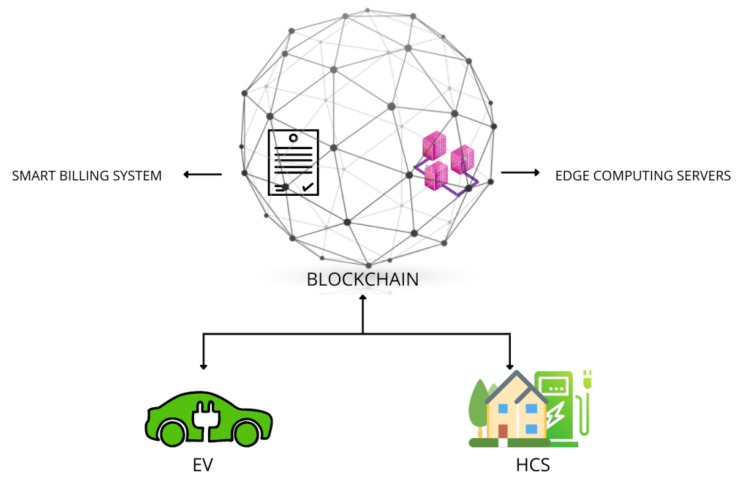
Components of the system.

In Figure 2, visual representations of the components are given.

#### 4.1.1. Electric Vehicles (EV)

Vehicles that can utilize electricity (completely or partially) to store and later convert it to kinetic energy are known as EVs. In the proposed system, any EV can register with the system and get a charging facility from the registered HCS. To register with the system, a person with an EV charging facility has to provide their national identity information. The system will perform verification of the ID, phone number, and email address before accepting the person as a member. After receiving any service, EVs can send their feedback related to the service provider, and their ratings will be published publicly, which will help the EVs select the best service provider nearby.

#### 4.1.2. Home Charging Stations (HCS)

A home charging station (HCS) is a station that is owned by an individual who may have an EV (or several), and that station is used to charge personal EVs or can be offered as a charging station (as a service) to other EVs commercially. HCS owners are also required to register with the system by providing detailed information: identity, charging equipment, facilities, capabilities, service time, location, etc. All the information is visible to the potential user before the decision to select the service. All the HCSs can choose their own pricing per kilowatt (KW), and the EV that selects a particular HCS will agree to that price. Every HCS will receive a rating point after providing any service, and the rating points will be available online. It will help the HCSs maintain the quality of service and the pricing level. Too-costly services may be given poor ratings by the service users (i.e., EVs’ owners who have used the service).

#### 4.1.3. Smart Billing System (SBS)

To calculate the amount of charge transferred from an HCS to an EV, a proper measurement system is used named SBS. SBS calculates the amount of charge in kilowatts (KW) and determines the amount to be paid according to the price asked by the HCS. In the proposed system, a smart meter is used to calculate the amount of charging, and the HCS will share it using the blockchain. As members of the blockchain, all the EVs and HCSs are connected to the blockchain by using a cryptocurrency. Once all calculations are done and fixed, the amount will be automatically deducted from the EV’s account and then credited to the account of the HCS.

#### 4.1.4. Blockchain

To join the proposed system, interested components (EVs, HCSs) are required to be registered by providing the necessary information and documentation. All the members will receive a pair of keys (public and private). The public key will be used as the member’s identity, and all the communications will take place by using that. At the same time, the public key will hide the real identity of the member, and in this way, it can protect privacy as well. However, a typical blockchain has to go through a lot of complex calculations because of block generation and validation, which would require servers with high computational capabilities. Thus, an edge server is used to perform those calculations to minimize delays during transactions. With the authentication information, the blockchain is also responsible for storing all the outcomes of smart billing and reputation management system inside a transaction to ensure their security, integrity, availability, transparency, etc.

**Figure 3:**
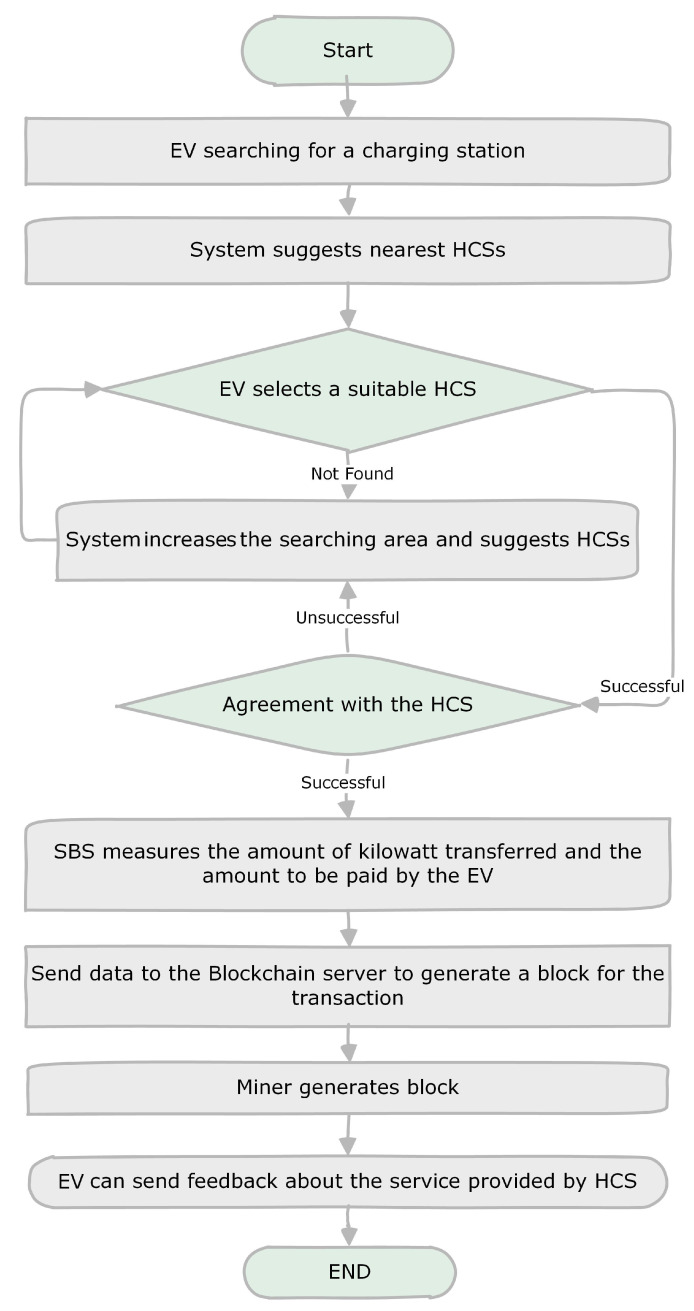
Workflow of the proposed system.

Any EV or HCS can register with the system by providing the required information and documents. After registration, it becomes a member of the blockchain and is able to perform transactions anytime over an Internet connection. An EV user that wants to charge its car can send a request for charging. Then, the system will suggest the nearby HCSs. Two factors will be applied while suggesting HCSs; one is the distance from the EV, and the other is the ratings of the HCSs. The EV can select the most suitable HCS among those on offer. When there is a mutual agreement between an EV and an HCS, the system will generate an ID for the transaction. In the future, information related to that charging will be identified uniquely by using that same ID. After receiving the service from an HCS, the SBS will calculate the amount of energy (i.e., the amount of charge) transferred and send a transaction to the blockchain to store the information. At the same time, the system will deduct the payment from the EV and move it to the HCS’s account. The flow of the system is illustrated in Figure 3.

### 4.2. Communication System

**Figure 4:**
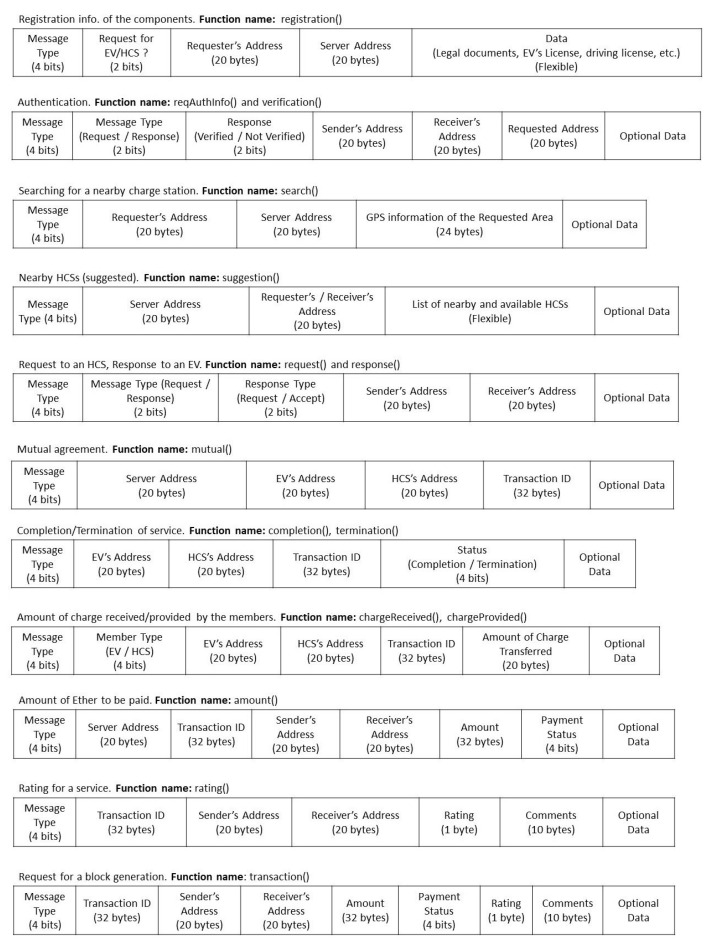
Message Formats.

To manage all the underlined protocols, several messages are passed among the blockchain’s components and the servers. The message formats are presented in Figure 4. All the messages start with the *message type* field, which informs user about the kind of information that resides inside the message and what possible actions are required to be taken.

According to the requirements, addresses, i.e., public keys, of EVs, HCSs, and servers, are added. However, to understand the format more clearly, sender/requester and receiver are shown on the figure. Completion and termination functions also do similar operations.

Some packet formats are applicable for multiple functions. For example, the same format is used for the *request()* and *response()* functions, where there is a field that informs the receiver about the type of message (request/response) and the status of the message. All the messages have a special field called optional data, which can be used for different purposes. Which function is used by which entity is presented in Table 2.

### 4.3. Authentication Protocol

In the proposed system, blockchain is used to confirm the authenticity of the members, i.e., EVs and HCSs. All of them are required to be registered physically before getting services from the system. They receive a pair of keys after the registration, and later all the communications will take place with their public keys. Before generating any request of charging, the system checks the membership status of the EVs, and similarly, before suggesting nearby HCSs, the system checks the authenticity of the HCSs. Moreover, to ensure the authenticity of a particular EV or HCS, any of the members can send a request for authentication information of another component by sending a message to the server by using *reqAuthInfo()*. Then, the server will reply with the authenticity of the requested components. In this way, the authenticity of the components is ensured so that both parties can initiate a safe and secure connection. Moreover, using the public keys instead of real identities will protect their original identities and privacy.

### 4.4. Smart Billing System

**Figure 5:**
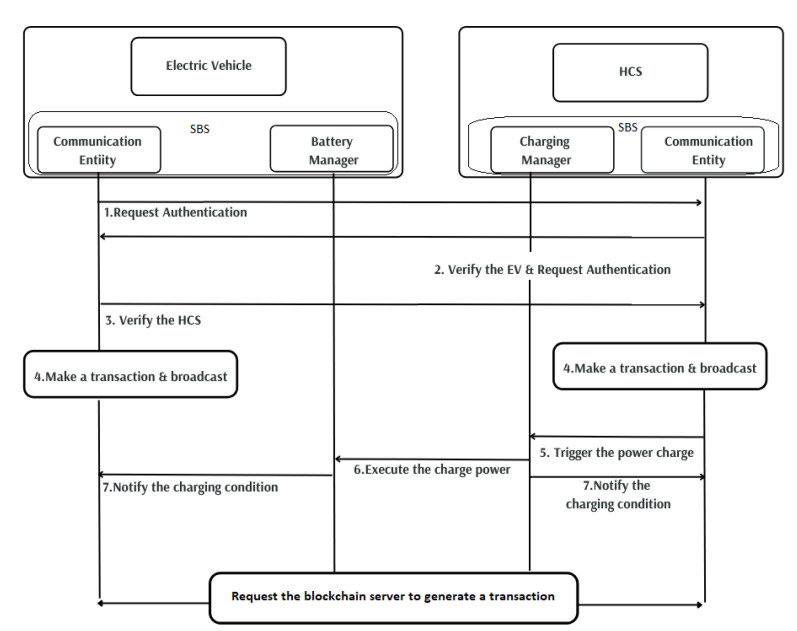
Operational flow of the billing system process.

The billing system for electric vehicles is a central hub that manages the exchange of electricity between the charging station and the EV. This proposed smart billing system (SBS) has two entities: a power management entity and a communication entity. The communication entity can also exchange information with other entities using wireless communication. The power management entities reside inside both EVs and HCS. This entity calculates and reports the amount of power to be charged from both sides. After the charging is finished, SBS verifies the amount of charge that has been transferred and sends it to the blockchain server as a transaction. The operational flow of charging and billing is illustrated in Figure 5.

### 4.5. Reputation Management System

A reputation management system was added to the system to ensure the quality of service. After receiving the charging service, the EV user can leave feedback about the received service. A rating from one to five can be provided, where five (5) indicates the best service and one (1) means the service is the worst possible. The server will calculate the mean of overall ratings for each HCS, and when suggesting nearby HCSs to the EVs, HCSs with relatively higher ratings will get priority on the list. Additionally, the EV users will be able to provide a comment describing the service received. After receiving the feedback from the EV, all the fields of the transaction are completed, and the server will generate the block from the transaction.

This will help HCSs to decide on their pricing, as they have the authority to fix their service rates; an HCS with a higher rating may ask for a higher price, and a newcomer may ask for a lower price to get good review scores. In this way, the reputation system will also be useful to create competition between the service providers, i.e., HCSs.

## 5. Implementation

To emulate the proposed blockchain-based P2P EV charging system, a virtual environment was created. Several virtual machines were prepared to represent EV, HCS, and blockchain servers. It was assumed that a specific amount of charge was transferred from an HCS to an EV, and the SBS requested a transaction in the blockchain. To simulate the blockchain, a blockchain testing platform called *Truffle* was used [43]. This platform provides a real blockchain with smart contract programming facilities. It provides *Ganache* [44], which simulates a real dummy of the Ethereum blockchain and additionally provides programming ability, customization, monitoring, debugging facilities, etc. The smart contract was written in Solidity programming language and deployed using Truffle. To develop the client side, a lightweight node server [45] was used with Node Packet Manager (NPM) [46].

The target of the implementation was to simulate the transactions, blockchain-based operations such as block generation, and cryptocurrency-based payment management in a real-world environment. Thus, the Ethereum blockchain was selected as the blockchain, and *Ether* as the cryptocurrency. However, some of the transactions were not simulated to simplify the experimental analysis.

### 5.1. Experimental Setup

The following steps can explain the experimental setup:

- For the experiment, multiple virtual machines were used via VM VirtualBox 6.1. Four VMs were set up: two of them represented EVs (named *EV${}_{1}$* and *EV${}_{2}$*), and the other two were HCSs (named *HCS${}_{1}$* and *HCS${}_{2}$*).
- Another one was set up in the blockchain server named *BCS*. Truffle platform and the Ganache blockchain were set up in the *BCS*. Moreover, for web hosting and management, a lightweight node server [45] and Node Packet Manager (NPM) were used.
- All the EVs and HCSs were considered as full members of the blockchain, and in *Ganache*, they were registered. Before beginning, 100 were assigned virtual Ether, which is the currency used by the Ethereum blockchain.
- EVs and HCSs use Metamask [47] as an Ethereum wallet, by which they can connect to the blockchain. Simultaneously, EVs can pay and HCSs can receive money.
- The communication module of the SBS is prepared for the experiment, and to simplify the experiment, instead of the charge measurement system, the amount of charge transferred from HCS to EV was assumed.
- The *Truffle* framework supports multiple smart contract programming languages. In this experiment, *Solidity* programming language was used to manage communication, block generation, and so on. For each and every activity, a function is responsible. Details of the functions are shown in Table 2. The structure of the transferred messages is illustrated in Figure 4.
- EVs can provide feedback after getting services from HCSs, which would be helpful in maintaining the quality of services.

### 5.2. Deploying the Blockchain

To run the experimental setup, firstly, the *Ganache* blockchain was deployed in the *BCS* machine. By default, *Ganache* generates some public keys for users, and all the users receive 100 *Ether* transactions. Each member VM (EV${}_{1}{,}_{2}$, HCS${}_{1}{,}_{2}$) got a public key and used that as its public identity. Then, the members joined the blockchain by using the *Metamask* wallet.

During development, we kept the amount of charge open to receive manual entry so that it could receive user input rather than automatic calculation by the charging agent. After deploying the blockchain, we requested different amounts of charging in KWs manually. After receiving the entry from the members, the SBS module generated the amount to be paid and requested a blockchain transaction. Due to the simplification, the proposed system can generate the block almost instantly after the request and broadcast it to all the members. During the block generation process, the amount of cryptocurrency, i.e., *Ether*, is deducted from the EV’s account and credited to the HCS’s account. Additionally, the service receiver EV can provide a rating score (out of 5), and the server will calculate the mean of all the reputation scores received by each HCS. The rating score will be available publicly. By using the web interface, all the members can check the global (and also own) transaction histories, financial statements, and rating points (provided or received).

**Figure 6:**
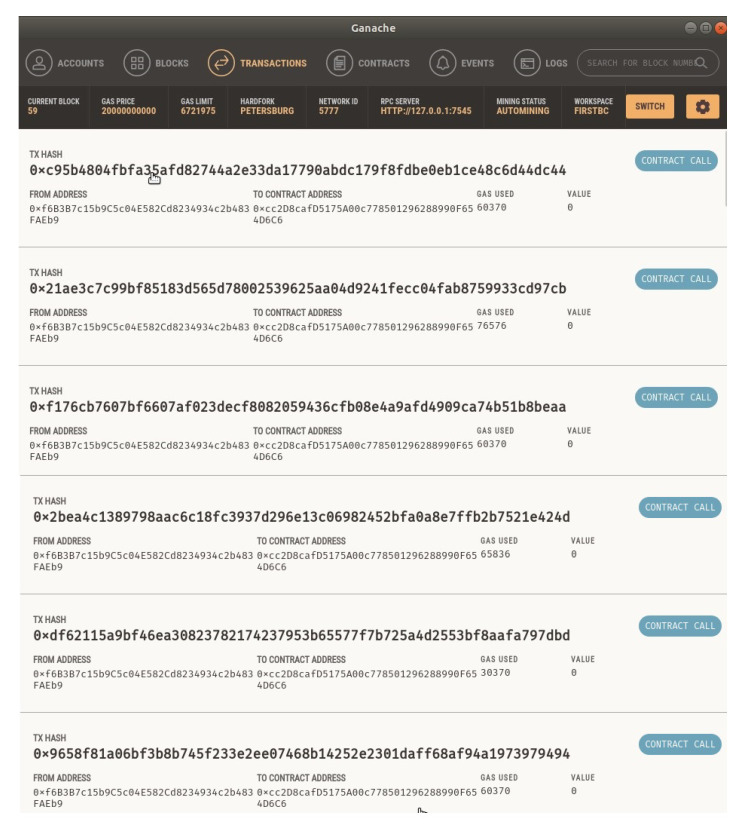
Transactions in the *Ganache* blockchain.

Multiple transactions were performed to analyze the system. After running the simulated system, data were prepared manually, and by using smart contracts, information was added to the *transaction()* function according to the message structure (presented in Figure 4). After inserting those data, a transaction was performed in the blockchain. Details of some of the performed transactions can be found in Figure 6.

## 6. Performance Analysis

Our method aims to implement a blockchain-based P2P charging system where the payments will be exchanged using the modern money exchange solution called cryptocurrency. Due to that, the performance analysis section presents the feasibility analysis and the advantages that can be achieved from this proposed method.

### 6.1. Storage Overhead

In a typical Ethereum blockchain, near 2KB are required per transaction, and a block can accept 512 transactions per block [40]. The average block size for Ethereum is 83.557 KB [48]. Thus, each 512 charge exchange transaction will require almost 84KB of storage.

### 6.2. Computational Time

**Figure 7:**
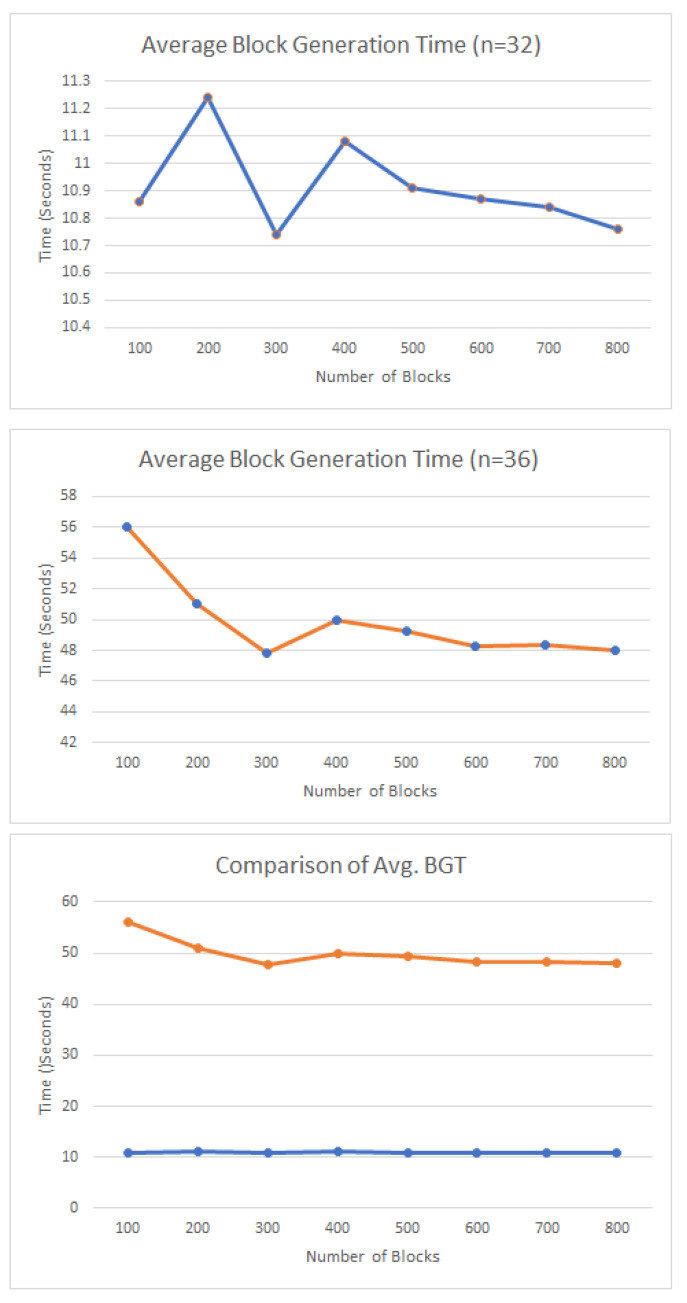
Average block generation time (BGT) when the difficulty of the consensus is 32 or 36, and a comparison.

Elliptic Curve Cryptography (ECC) is the algorithm used in Ethereum, which is one of the strongest algorithms against cryptanalysis [49]. Another factor that consumes time is the consensus protocol. Ethereum generally uses the Proof-of-Work (PoW) method as consensus. If the consensus is PoW and the cryptography algorithm is ECC, it requires about 4 min to generate 40 blocks, and the difficulty is 32.49 KH [50]. The average block generation times with different difficulties [50] are presented in Figure 7 (*n* is the difficulty of the consensus protocol).

### 6.3. Propagation Time

As the proposed method is designed to provide remote support, it requires propagation time to be minimized to maintain the efficiency of the system. However, today’s high-speed Internet connections are sufficient to provide necessary services, i.e., reasonable propagation time. For example, by using a 5G internet connection, it is possible to transmit 50 Mbps to 1 Gbps, but the 6G connection will make it at least 100 times faster than that [51]. Thus, the main two components of the system, i.e., EVs and HCSs, are required to maintain a high-speed Internet connection to maintain high throughput.

## 7. Challenges and Limitations

Blockchain was first proposed in 2008, and it was utilized mainly to develop a currency exchange system for some time [13]. Later in 2014, Ethereum came up with the concept of smart contracts, which influenced researchers to utilize blockchains in different fields [52,53]. In fact, that worked like a catalyst for the creation of innovative applications and areas where they could be used. As a newly developed system, it still requires more experiments (and convincing proofs) to make it compatible with other systems.

The proposed method can also be considered as an effort to combine the P2P energy exchange with blockchain technology. Thus, while developing such a system, several challenges were faced. First of all, it is really difficult to develop a real-world system that can directly communicate with a blockchain. Thus, a simulation study was performed where VMs were considered EVs and HCSs. Secondly, there are very few way to learn and develop smart contracts. Thirdly, rather than popular languages, it supports newly developed languages such as Solidity, Serpent, and Yul, which makes the development phase more difficult. However, some simplified code was written using Solidity (which is an object-oriented, high-level language for implementing smart contracts) to simulate the proposed method. In spite of our efforts, it still requires improvement and optimization. Thirdly, to ensure the trustability of the blockchain, our mechanism uses a consensus protocol which could be highly time consuming (at times), and thus, it is considered a barrier while developing a system that requires high throughput.

While proposing our system, we have considered this above-mentioned issue about time consumption, and accordingly developed the system in such a way that the slowness of the block generation process would not harm the ongoing flow of the system. None of the components and none of the protocols have to wait for block generation, but rather, they can just perform the required actions. After all the communications, tasks, and transactions (starting from *searching()* and ending with *feedback()*) are completed, the server initiates the block generation process. Before the block generation, the charging process and money calculation are performed. Hence, EVs can leave the site just after receiving the charges. As the payment is done after the block generation, the HCSs are required to wait. However, as the system is secure using the blockchain, there is no confusion left regarding receiving the payment and getting extraordinary services, such as security, integrity, transparency, and availability; and HCSs can endure the delay and identify the service taker. We have mentioned the registration system under the blockchain to identify who would be allowed to get the service. While this gives the solution to this issue, a more efficient method can be searched for to enhance the performance of the blockchain. In the future, we plan to also find out more suitable ways to increase the throughput and more optimized outcomes from the currently designed system.

As blockchain-based systems have become popular recently, they were not developed to support all kinds of systems (just yet). Hence, there are several practical issues while adapting blockchain to new systems. Scalability is a critical limitation of the blockchains because of their decentralized and distributed nature. As all the members store the block information, the system requires a huge amount of storage space compared to a typical centralized system. Again, to ensure security, several cryptographic functions are required to be used for encryption, decryption, hashing, etc., which would require high computational power, and that would make the blockchain system expensive. Moreover, systems with low computational power require substantial computational time, which minimizes the throughput of the system. However, to mitigate these problems, several online solutions, such as edge/fog computing, would help the blockchain-based system, as they can virtually provide high computational and storage capacity and can perform complex cryptographic calculations in short periods of time.

## 8. Conclusions and Future Direction

With the gradual increase in environment-friendly electric vehicles, the availability of charging stations should be ensured. However, it is not an easy task anyway to make them available everywhere, especially in rural areas. Our proposed method largely solves this issue, as it uses an approach of enhanced peer-to-peer charging of EVs, which would increase the availability of charging stations without much change to the existing infrastructure. It will use the same areas but will employ a mechanism to make bonds between the EVs and charging stations anywhere. The rating system can be very useful for keeping the market prices in check and ensuring the quality of the service.

The transactions without a third party are made safe via integrated blockchain to secure the environment for the member nodes of the blockchain for trade. Moreover, a cryptocurrency-based payment system makes the system easy, automated, hassle-free, time-saving, durable, environment-friendly, immutable, and available worldwide. Furthermore, enhanced technological support structures, such as edge computing, and high-speed 5G/6G Internet, can be easily combined with the system to make it more efficient. In the future, we plan to integrate the proposed system with real EVs and HCSs and collect real-world data from those, and make necessary arrangements to enhance the quality of service.

## Figures and Tables

**Table 1:** Comparison of the Ethereum blockchain with others.

Blockchain	Symbol	Scripting Language	Implementation Language	Average TPS ${}^{1}$	SC ${}^{2}$ Support
Ethereum	ETH	Solidity, Serpent, LLL	Go-Ethereum, CPP-Ethereum, Py-Ethereum, EthereumJ, Parity	5.40	Yes
Bitcoin	BTC/XBT	Bitcoin Script	C++	3.50	No
Zcash	ZEC	Bitcoin Script	C++	0.06	No
Litecoin	LTC	Bitcoin Script	C++	0.35	No
Dash	DASH	Bitcoin Script	C++	0.07	No
Peercoin	PPC	Bitcoin Script	C++	0.01	No
Ripple	XRP	N/A	C++	10.75	No
Monero	XMR	N/A	C++	0.06	No
MultiChain	-	Bitcoin Script	C++	1000	No
Hyperledger	-	Go, Node.js, Java, C++, Python and more	Go, Python and More	Various	Yes

${\;}^{1}$ TPS = transactions per second. ${\;}^{2}$ SC = smart contract.

**Table 2:** Smart contract functions in the proposed system.

Responsibility	Function	Performed By		
	Name	EVs	HCSs	Server
Registration info. of the components	registration()	✓	✓	-
Request for authentication info.	reqAuthInfo()	✓	✓	-
Verification of Authenticity	verification()	-	-	✓
Searching for a charge station nearby	search()	✓	-	-
Nearby HCSs (suggested)	suggestion()	-	-	✓
Request to an HCS	request()	✓	-	-
Response to an EV	response()	-	✓	-
Mutual agreement	mutual()	✓	✓	✓
Completion of service	completion()	✓	✓	-
Termination of a service	termination()	✓	✓	-
Amount of charge received by EV	chargeReceived()	✓	-	-
Amount of charge provided by HCS	chargeProvided()	-	✓	-
Amount of *Ether* to be paid	amount()	-	-	✓
Rating for a service	rating()	✓	-	-
Request for a block generation	transaction()	-	-	✓
